# Identification of Activated Protein Kinase Cα (PKCα) in the Urine of Orthotopic Bladder Cancer Xenograft Model as a Potential Biomarker for the Diagnosis of Bladder Cancer

**DOI:** 10.3390/ijms22179276

**Published:** 2021-08-27

**Authors:** Takahito Kawano, Yoko Tachibana, Junichi Inokuchi, Jeong-Hun Kang, Masaharu Murata, Masatoshi Eto

**Affiliations:** 1 Center for Advanced Medical Innovation, Kyushu University, 3-1-1 Maidashi, Higashi-ku, Fukuoka 812-8582, Japan; t-kawano@dem.med.kyushu-u.ac.jp (T.K.); y_tachi@camiku.kyushu-u.ac.jp (Y.T.); 2 Department of Urology, Graduate School of Medical Sciences, Kyushu University, 3-1-1 Maidashi, Higashi-ku, Fukuoka 812-8582, Japan; 3 Division of Biopharmaceutics and Pharmacokinetics, National Cerebral and Cardiovascular Center Research Institute, 6-1 Shinmachi, Kishibe, Suita, Osaka 564-8565, Japan

**Keywords:** protein kinase Cα, bladder cancer, cystoscopy, urinary biomarker, orthotopic xenograft mice

## Abstract

Bladder cancer has a high recurrence rate; therefore, frequent and effective monitoring is essential for disease management. Cystoscopy is considered the gold standard for the diagnosis and continuous monitoring of bladder cancer. However, cystoscopy is invasive and relatively expensive. Thus, there is a need for non-invasive, relatively inexpensive urinary biomarker-based diagnoses of bladder cancer. This study aimed to investigate the presence of activated protein kinase Cα (PKCα) in urine samples and the possibility of PKCα as a urinary biomarker for bladder cancer diagnosis. Activated PKCα was found to be present at higher levels in bladder cancer tissues than in normal bladder tissues. Furthermore, high levels of activated PKCα were observed in urine samples collected from orthotopic xenograft mice carrying human bladder cancer cells compared to urine samples from normal mice. These results suggest that activated PKCα can be used as a urinary biomarker to diagnose bladder cancer. To the best of our knowledge, this is the first report describing the presence of activated PKCα in the urine of orthotopic xenograft mice.

## 1. Introduction

The 5-year relative survival rate for bladder cancer is more than 77% for all disease stages. Most diagnosed bladder cancers are non-muscle-invasive, with a favorable survival rate of 70–80%. However, the recurrence rate of non-muscle-invasive bladder cancer can be as high as 50–90% [1,2,3,4]. Moreover, recurring non-muscle-invasive bladder cancer progresses to muscle-invasive bladder cancer, which has a 5-year survival rate of less than 50%. Therefore, predicting a patient’s risk of recurrence and progression is essential for appropriate treatment.

The most commonly used diagnostic tests for bladder cancer are urine cytology and cystoscopy. Urine cytology, which is non-invasive and relatively inexpensive, exhibits high specificity, and high sensitivity to high-grade bladder cancer; however, it has very low sensitivity to low-grade bladder cancer, at less than 30% [2,4,5,6]. On the other hand, cystoscopy, considered to be the gold standard for detecting bladder cancer, achieves relatively high detection rates for low-grade and high-grade bladder cancers, despite its relatively low success in detecting flat lesions such as bladder carcinoma in situ. However, cystoscopy is invasive and relatively expensive; in addition, it can cause pain and discomfort, more so in men than in women [2,7,8].

In contrast, the urinary biomarker-based detection of bladder cancer is both non-invasive and relatively inexpensive [9]. The Food and Drug Administration (FDA) has approved six urinary biomarker tests for the diagnosis of bladder cancer: quantitative nuclear matrix protein 22 (NMP22; Alere NMP22 Test), qualitative NMP22 (NMP22BladderChek), qualitative bladder tumor antigen (BTA; BTA stat test), quantitative BTA (BTA TRAK), fluorescence in situ hybridization (UroVysion), and fluorescent immunohistochemistry (ImmunoCyt) [10]. However, many urinary biomarkers, including those in FDA-approved tests, are relatively poor indicators of low-grade bladder cancers, at 32–75%, and are relatively effective indicators of high-grade bladder cancers, at 47–85% [5,9,10]. Therefore, there is a need for more sensitive and specific urinary biomarkers for the diagnosis of bladder cancers. Recent advances in next-generation sequencing and other assays have led to the development of new urinary biomarkers targeting mRNA, DNA mutation, methylation and microRNAs. Although good results in sensitivity and specificity have been reported, they are expensive and have not yet been approved by the FDA [11].

Protein kinase C (PKC) signaling is critical in controlling the expression of genes related to cell cycle progression and cancer formation and development. PKC, a family of phospholipid-dependent serine/threonine kinases, can be classified into three subfamilies based on their structural and activation characteristics: conventional or classic PKC isozymes (cPKCs; α, βI, βII, and γ), novel or non-classic PKC isozymes (nPKCs; δ, ε, η, and θ), and atypical PKC isozymes (aPKCs; ζ, ι, and λ) [12,13]. The activation of cPKCs requires diacylglycerol (DAG) as an activator and phosphatidylserine (PS), and Ca^2+^ as cofactors. The nPKCs are regulated by DAG and PS; however, they do not require Ca^2+^ for activation. Meanwhile, the activity of the atypical PKCs is stimulated only by PS and not by DAG or Ca^2+^, or both [12,13,14].

PKCα activation is involved in the development and progression of multiple types of cancers through its inhibition or stimulation of various cellular signaling pathways [12,14]. For example, the level of activated PKCα is elevated in bladder cancer cells and tissues; in addition, activated PKCα is involved in bladder cancer cell proliferation, survival, invasion, migration, and anticancer drug resistance [13,15,16,17]. Furthermore, our group reported that activated PKCα in blood and tissue samples could be a useful biomarker for cancer diagnosis [18,19,20,21]. However, whether activated PKCα can be used as a urinary biomarker remains to be determined.

This study aimed to investigate whether activated PKCα could be a urinary biomarker for the diagnosis of bladder cancer. This possibility was examined using human bladder cancer cell lines and urine samples collected from mice of the orthotopic bladder cancer xenograft model.

## 2. Results

### 2.1. Activated PKCα in Human Bladder Cancer Cells

**Figure 1:**
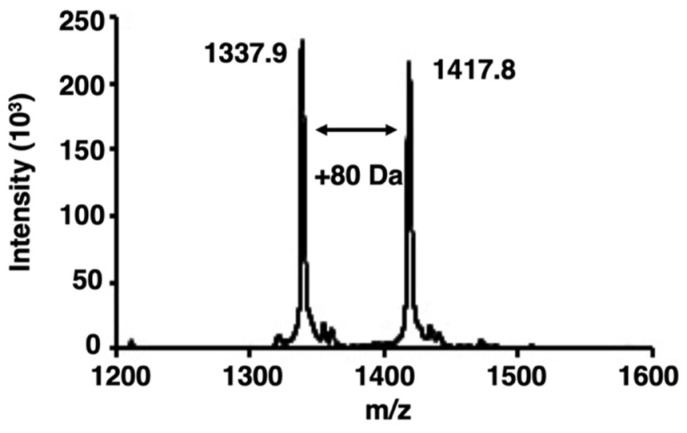
Matrix-assisted laser desorption/ionization–time-of-flight mass spectrometry (MALDI-TOF MS) spectra of phosphorylation by protein kinase Cα (PKCα). The spectra were obtained from the phosphorylation reaction of a PKCα-specific peptide substrate with the lysate of bladder cancer cells. The peak of the phosphorylated peptides showed an increase of 80 Da.

We examined whether bladder cancer cells contained activated PKCα by analyzing the phosphorylation of a PKCα-specific peptide substrate [22] using matrix-assisted laser desorption/ionization–time-of-flight mass spectrometry (MALDI-TOF MS). When peptides or proteins are phosphorylated by target protein kinases, the phosphorylated peptides or proteins are identified by the appearance of a new peak with an additional mass of 80 Da [23]. Here, an increase of 80 Da in the *m*/*z* value was observed in the PKCα-specific peptide after phosphorylation (Figure 1).

**Figure 2:**
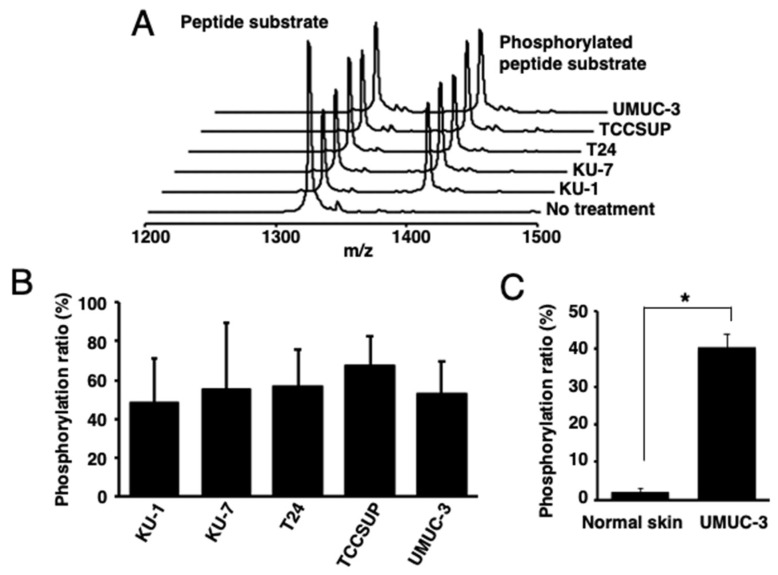
Identification of activated PKCα in human bladder cancer cells. (**A**) The MALDI-TOF MS spectra and (**B**) phosphorylation ratios after the phosphorylation of a PKCα-specific peptide by the lysates of five human bladder cancer cell lines. (**C**) Phosphorylation ratios after the phosphorylation of a PKCα-specific peptide substrate with the lysate from cancer cell (UMUC-3)-bearing mouse tissues or normal mouse tissues (n = 3). * *p* < 0.01.

Then, the PKCα-specific peptide was reacted with lysates of human bladder cancer cells [KU-1 (grade 2), KU-7 (grade 1), T24 (grade 3), TCCSUP (grade 4), or UMUC-3 (grade 3)], tissues from UMUC-3-bearing mice, or normal skin tissues. Higher phosphorylation ratios were observed in all the reactions using human bladder-cancer-derived cells and tissues than those with normal skin tissues (Figure 2A–C).

**Figure 3:**
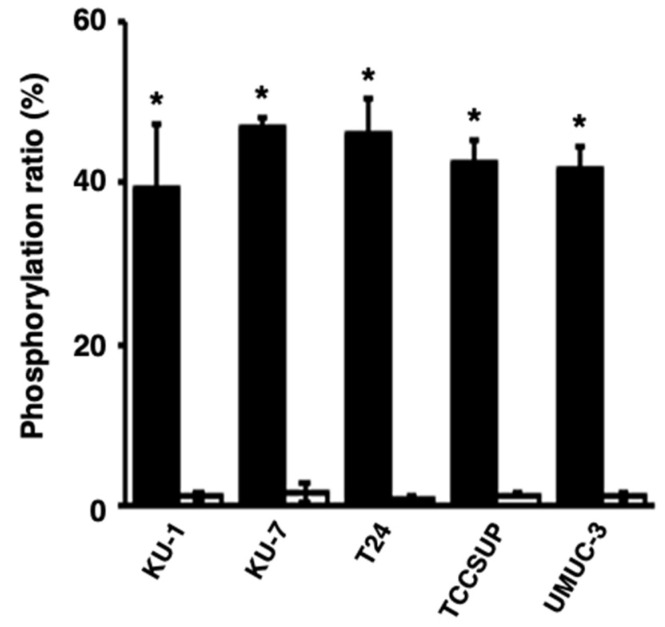
Release of activated PKCα from human bladder cancer cells. The phosphorylation ratios of the phosphorylation of a PKCα-specific peptide by the medium of bladder cancer cells after a 24 h culture (black bars) or medium only (white bars) were calculated. * *p* < 0.01, versus medium only.

Next, we studied whether activated PKCα could be released from bladder cancer cells and whether the released PKCα could phosphorylate the PKCα-specific peptide. The phosphorylation level of the peptide in reactions with the culture media of the TCCSUP and UMUC-3 cells was significantly higher than that with medium only (Figure 3), strongly suggesting that activated PKCα was released from bladder cancer cells and its activity was maintained.

### 2.2. Activated PKCα in the Urine of Orthotopic Xenograft Mouse Models of Human Bladder Cancer

**Figure 4:**
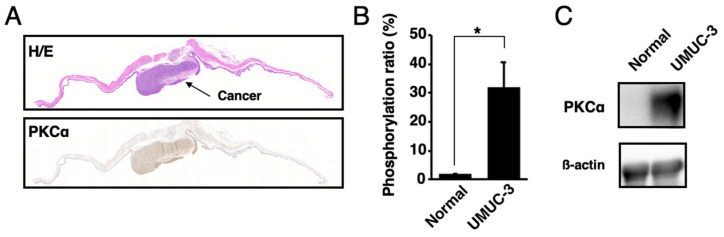
Localization of activated PKCα in bladder cancer tissues and normal bladder tissues. (**A**) Histological analysis of the bladder cancer tissues or normal bladder tissues in the orthotopic xenograft mice with UMUC-3 cells. Tissue sections were stained with hematoxylin–eosin (H/E) and immunohistochemically stained with anti-phosphoPKCα. (**B**) Phosphorylation ratios and (**C**) Western blot analysis of the lysates from bladder cancer tissues or normal bladder tissues (n = 3). * *p* < 0.01.

The presence of activated PKCα in urine samples was analyzed. The orthotopic xenograft mouse model of human bladder cancer was established by the direct injection of UMUC-3 cells into the mouse bladders. According to the Western blot, MALDI-TOF MS, and histological analysis, activated PKCα was present at higher levels in bladder cancer tissues than in normal bladder tissues (Figure 4 and Figure S1).

**Figure 5:**
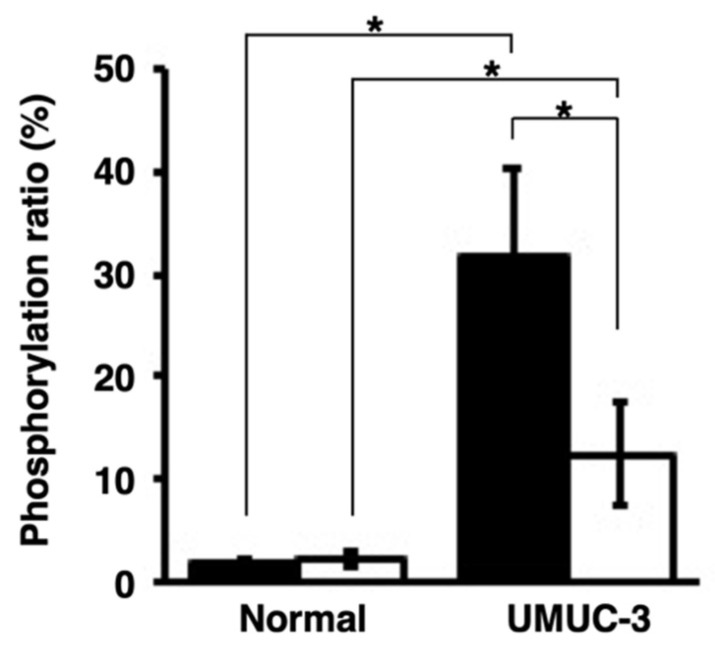
Detection of activated PKCα in the urine samples collected from orthotopic xenograft mice with UMUC-3 and normal mice (n = 3). The phosphorylation ratios were calculated after phosphorylation of the PKCα-specific peptide with the urine sediments (black bars) or urine supernatants (white bars). * *p* < 0.01.

Next, urine samples were collected from the orthotopic xenograft mice, and the peptide was reacted with the urine supernatants or sediments. Peptide phosphorylation was detected. Higher phosphorylation ratios were observed in the reaction with the urine sediments containing exfoliative bladder cancer cells than with the urine supernatants. On the other hand, extremely low phosphorylation ratios were observed in the phosphorylation reactions with the urine supernatant and sediment from normal mice (Figure 5). These data suggest the presence of activated PKCα in urine samples.

## 3. Discussion

MALDI-TOF MS is a useful tool for analyzing peptide phosphorylation. However, cancer cell and tissue lysates containing variable biomolecules, such as proteins and lipids, can prevent crystal formation and interfere with the ionization of the α-Cyano-4-hydroxycinnamic acid matrix (CHCA) matrix and lysate samples at a 1:1 volume ratio, resulting in low signal intensity [24,25]. These problems were overcome by using a matrix-to-sample ratio of 100:1 to detect the phosphorylated peptide. The concentration of peptide, at 0.3 μM, at the 100:1 ratio was within detection ranges for MALDI-TOF MS at 10 nM to 100 μM [24]. Thus, satisfactory signal intensities could be obtained.

PKCα is hyperactivated in the cells and tissues of several cancers, including bladder cancer [13,15]. Previous studies have reported that PKCα expression grade-dependently increased in cancer tissues from patients with bladder cancer, but was identified in low and high grades [26,27,28]. Furthermore, PKCα is expressed at higher levels in bladder cancer tissues than in normal bladder tissues [29]. The bladder cancer cell lines used in this study represent different pathological grades, with KU-7 for grade 1, KU-1 for grade 2, T24 and UMUC-3 for grade 3, and TCCSUP for grade 4 [29,30,31]. However, activated PKCα was found in all the cell lines, and all the cell lysates phosphorylated a PKCα-specific peptide. These results indicate that activated PKCα can be a biomarker for the diagnosis of low- as well as high-grade bladder cancers.

Cancer diagnosis using liquid biopsies, e.g., blood, saliva, or urine, instead of tissue biopsies, offers several advantages. For example, liquid biopsies involve relatively easy and simple sampling and non-invasive methods which reduce the pain and risk for the patient. Previously, our group first reported the presence of activated PKCα in the blood taken from cancer-bearing mice [17,20] and patients with lung cancer [20]. The diagnostic accuracy for lung cancer by serum-activated PKCα was comparable to that of other lung cancer biomarkers, such as ProGRP, CEA, and CYFRA 21-1 [20].

In this study, activated PKCα was observed in the urine collected from the mice of the orthotopic xenograft model of human bladder cancer, but not in the urine from normal mice. Furthermore, higher levels of phosphorylated peptides were observed in the phosphorylation reactions with the urine sediments than those with the urine supernatants. Urine sediments contain exfoliative bladder cancer cells; thus, they may be more sensitive in diagnosing bladder cancer than urine supernatants. To the best of our knowledge, this study is the first to describe the presence of activated PKCα in the urine of orthotopic xenograft mice and demonstrates that activated PKCα can be used as a urinary biomarker for the diagnosis of bladder cancer.

However, the mechanism underlying the release of activated PKCα into the urine remains unclear. The activation of PKCα in the cell membrane may be related to its release into the urine. For example, whereas activated protein kinase A (PKA) remains in the cytosol of cancer cells, its N-myristoylation induces its interaction with the cell membrane. This interaction plays a critical role in the secretion of PKA into the extracellular space of cancer cells [32]. On the other hand, the translocation of PKCα from the cytosol to the membrane is crucial for its interaction with its activator DAG and cofactors, PS and Ca^2+^, which lead to PKCα activation [12,13,14]. In this study, the experiment with cell culture supernatants showed that activated PKCα was released into the extracellular space of bladder cancer cells. Therefore, the activated PKCα in the urine of orthotopic xenograft mice may be derived from the interaction of PKCα with the membrane of bladder cancer cells.

## 4. Materials and Methods

### 4.1. Peptide Synthesis

A PKCα-specific peptide substrate (Figure S2) was synthesized and purified as described previously [22]. This peptide exhibited a high affinity for various cancer cells and tissues but extremely low affinity for normal cells and tissues [18,19,22,33]. The purity of the peptide was verified using high-performance liquid chromatography and MALDI-TOF MS; a peptide sample with more than 95% purity was used for the phosphorylation reactions.

### 4.2. MALDI-TOF MS Analysis

α-Cyano-4-hydroxycinnamic acid matrix (CHCA) at 10 mg/mL was prepared in 50% water/acetonitrile and 0.1% trifluoroacetic acid. First, the matrix and samples were mixed at a ratio of 100:1. Then, 1 μL of the analyte/matrix mixture was applied to the MALDI plate and allowed to dry to induce crystallization. Analyses were conducted using a MALDI-TOF-MS autoflex speed (Bruker, Billerica, MA, USA) in the positive ion linear mode. All the spectra were analyzed using flexAnalysis (Applied Biosystems, Waltham, MA, USA). The phosphorylation ratio, defined as the ratio of the ion intensity of the phosphorylated material to that of the unphosphorylated material, was calculated using the following formula: [phosphorylated peptide intensity/(phosphorylated peptide intensity + non-phosphorylated peptide intensity) × 100].

### 4.3. Preparation of Cell Lysates

KU-1, KU-7, TCCSUP, and UMUC-3 cells (ATCC, Manassas, VA, USA) were maintained in low-glucose Eagle’s minimum essential medium (Wako, Osaka, Japan). T24 cells were maintained in RPMI-1640 medium (WAKO). The media were supplemented with 10% fetal bovine serum and 1% antibiotic/antimycotic mix (Gibco, Invitrogen Co., Waltham, MA, USA). The cells were incubated in a humidified atmosphere containing 5% CO_2_ at 37 °C. The cultured cells were removed by scraping and centrifuged for 5 min at 1500 rpm. After removing the supernatant, 0.2 mL lysis buffer containing 10 mM HEPES at pH 7.5, 250 mM sucrose, and cOmplete™ protease inhibitor cocktail (Roche, Tokyo, Japan) was added to the cells. The samples were sonicated for 10 s and centrifuged at 5000× *g* at 4 °C for 15 min; the resultant supernatant was used to phosphorylate the peptide substrate. The total protein concentration was determined using the Bradford protein assay (Coomassie Brilliant Blue G-250 reagent; Dojindo Laboratories, Kumamoto, Japan).

The release of activated PKCα from bladder cancer cells was investigated by incubating the cells at 37 °C for 24 h. Then, cell media were collected and phosphorylated with the PKCα-specific peptide.

### 4.4. Preparation of Tissue Lysates

All the animal care and experimental procedures, approved by the Committee on the Ethics of Animal Experiments, Kyushu University, were conducted following the Guidelines for Animal Experiments of Kyushu University. The mice were maintained in a 12 h light/dark cycle and provided with drinking water and food ad libitum. Six- to eight-week-old female Balb/c-nu/nu nude mice (Charles River Laboratories Japan, Yokohama, Japan) were used for the xenograft model. Mice (n = 3) were inoculated subcutaneously with 1 × 10^6^ UMUC-3 cells in 50 µL of Hanks’ balanced salt solution and 50 µL of Matrigel (Corning, Tewksbury, MA, USA). Tumors were allowed to grow to a mean diameter of approximately 1 cm. Cancers and normal skin tissues were excised and homogenized in lysis buffer containing 10 mM HEPES at pH 7.5, 250 mM sucrose, and cOmplete™ protease inhibitor cocktail. The samples were washed with buffer three times and sonicated for 30 s after adding 1 mL buffer. After sonication of the homogenate, the samples were centrifuged at 5000× *g* at 4 °C for 15 min, and the supernatant was used for phosphorylation reaction.

### 4.5. Preparation of Urine Samples from an Orthotopic Bladder Cancer Xenograft Mouse Model

After the mice (n = 6) were anesthetized with isoflurane, their bladders were pretreated with 100 μL of 0.1 mg/mL poly-L-lysine (Sigma-Aldrich, St. Louis, MO, USA) for 20 min through the urethra with vascular catheters (24 Gauge; Terumo, Tokyo, Japan). Then, the bladders were flushed with 100 μL of phosphate-buffered saline (PBS), and 5 × 10^6^ UMUC-3 cells in 50 μL of PBS were inoculated into the bladder. Before removing the catheters, the mice were placed under anesthesia for 4 h. The mice were divided into two groups: one group was used for urine collection (n = 3), and the other group was for histological and Western blot analyses and the phosphorylation reactions of bladder cancer tissues or normal bladder tissues (n = 3). At 4 weeks post-inoculation with UMUC-3 cells, urine samples were collected and centrifuged at 5000× *g* at 4 °C for 15 min. Then, the supernatants were transferred to new tubes. After adding 0.2 mL of lysis buffer, the samples were sonicated for 10 s and centrifuged at 5000× *g* at 4 °C for 15 min. The resulting supernatants were used for the phosphorylation reaction.

### 4.6. Phosphorylation of Peptide Substrate

The phosphorylation reaction was carried out in 30 μL of a buffer containing 10 mM HEPES at pH 7.5, 10 mM MgCl_2_, 100 μM ATP, and 30 μM synthetic peptide with cell lysates, a medium, or urine samples with 0.2 mg/mL protein. After incubation at 37 °C for 60 min, the samples were analyzed using MALDI-TOF MS. For each experiment, triplicate samples were prepared, and each sample was analyzed twice.

### 4.7. Western Blot

Equal amounts of proteins (20 µg/well) were separated by sodium dodecyl–sulfate polyacrylamide gel electrophoresis (Wako) and transferred to a membrane using the Trans-Blot Turbo Transfer System (Bio-Rad, Hercules, CA, USA). The membrane was washed three times with Tween-phosphate-buffered saline (PBS) (1×) and incubated with 5% skimmed milk in Tween-PBS (1×) for 60 min at room temperature to block nonspecific binding. The membrane was immunoblotted with anti-phosphoPKCα (Ser657; Abcam, Cambridge, UK) or anti-ß-actin antibody (Cell Signaling Technology, Inc., Danvers, MA, USA) at 4 °C overnight. The reacted proteins were detected with horseradish peroxidase-conjugated goat anti-rabbit IgG (1:10,000; Vector Laboratories, Burlingame, CA, USA) for 60 min at room temperature and visualized by chemiluminescence using the Clarity Western ECL Substrate (Bio-Rad).

### 4.8. Histological Analysis

The tumors were embedded in paraffin, sectioned, subjected to hematoxylin and eosin staining or immunostaining with antibodies against PKCα, and imaged using Axio Scan Z1 (Carl Zeiss AG. Ltd., Oberkochen, Germany)

### 4.9. Statistical Analysis

All results are expressed as the mean ± standard deviation. Statistical significance between groups was determined with the two-tailed Student’s *t*-test using Microsoft Excel Data Analysis (Microsoft, Redmond, VA, USA).

## 5. Conclusions

In this study, we investigated the presence of activated PKCα in urine and the possibility of PKCα as a biomarker for bladder cancer diagnosis. Activated PKCα was detected in human bladder cancer cells exhibiting different pathological grades, 1 to 4, and in the urine of orthotopic xenograft mice. These results suggest that activated PKCα can be used as a urinary biomarker for the diagnosis of bladder cancer.

## Data Availability

Data are contained within the article or Supplementary Materials.

## Supplementary Materials

The following are available online at https://www.mdpi.com/article/10.3390/ijms22179276/s1.
