# Supplementary material for: Identification of Activated Protein Kinase Cα (PKCα) in the Urine of Orthotopic Bladder Cancer Xenograft Model as a Potential Biomarker for the Diagnosis of Bladder Cancer
Source: Int J Mol Sci. 2021 Aug 27;22(17):9276. doi: 10.3390/ijms22179276 (PMC8430461; doi:10.3390/ijms22179276)
Supplement: Supplementary file 1 [file ijms-22-09276-s001.zip › ijms-1334715-SI.pdf]

Supplementary materials

Identification of activated protein kinase Cα (PKCα) in the urine of orthotopic bladder cancer xenograft model as a potential biomarker for the diagnosis of bladder cancer

Takahito Kawano, Yoko Tachibana, Junichi Inokuchi, Jeong-Hun Kang, Masaharu Murata, Masatoshi Eto

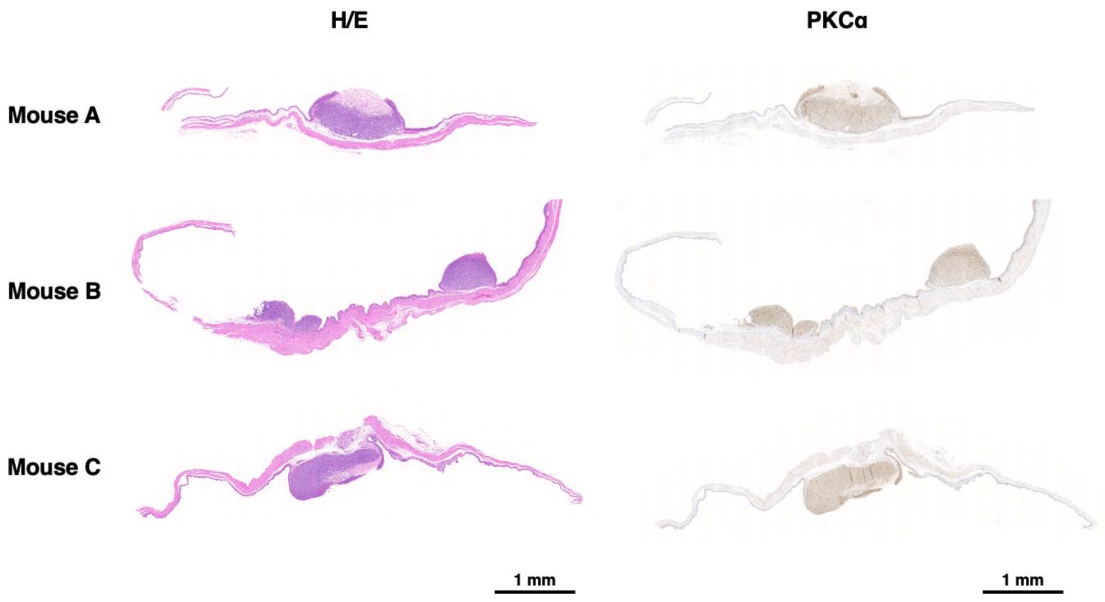

Figure S1. Histological analysis of the bladder cancer tissues in the orthotopic xenograft mice with UMUC-3 cells (n=3). Tissue sections were stained with hematoxylin–eosin (H/E) and immunohistochemically stained with anti-phosphoPKCα. Images of Mouse C were used in Figure 4A.

Ac-FKKQG***S***FAKKK-NH_2

Figure S2. The sequence of PKCα-specific peptide substrate used in this study. The peptide was N-terminally acetylated (Ac) and C-terminally amidated (NH_2). The phosphorylation site is marked in bold and italics. A, alanine; F, phenylalanine; G, glycine; K, lysine; Q, glutamine; S, serine.
